# Co‐producing research with youth: The NeurOx young people's advisory group model

**DOI:** 10.1111/hex.12911

**Published:** 2019-05-16

**Authors:** Gabriela Pavarini, Jessica Lorimer, Arianna Manzini, Ed Goundrey‐Smith, Ilina Singh

**Affiliations:** ^1^ Department of Psychiatry and Wellcome Centre for Ethics and Humanities University of Oxford Oxford UK; ^2^ Oxford Neuroscience, Ethics and Society Young People's Advisory Group Oxford UK

**Keywords:** bioethics, children's rights, co‐production, involvement, mental health, participation, public engagement, young people, young persons' advisory group

## Abstract

**Context:**

The 1989 UN Convention on the Rights of the Child states that children have the right to be heard in all matters affecting them. The Convention inspired a surge in research that investigates young people's perspectives on health and wellness‐related concerns and that involves children as ‘co‐researchers'. Young people's advisory groups (YPAGs) are a widely used method to enable young people's involvement in all research stages, but there is a lack of academic literature to guide researchers on how to set up, run and evaluate the impact of such groups.

**Objective:**

In this paper, we provide a step‐by‐step model, grounded in our own experience of setting up and coordinating the Oxford Neuroscience, Ethics and Society Young People's Advisory Group (NeurOx YPAG). This group supports studies at the intersection of ethics, mental health and novel technologies. Our model covers the following stages: deciding on the fit for co‐production, recruiting participants, developing collective principles of work, running a meeting and evaluating impact.

**Results:**

We emphasize that throughout this process, researchers should take a critical stance by reflecting on whether a co‐production model fits their research scope and aims; ensuring (or aspiring to) representativeness within the group; valuing different kinds of expertise; and undertaking on‐going evaluations on the impact of the group on both the young people and the research.

**Conclusion:**

Adopting a critical and reflective attitude can increase researchers' capacity to engage youth in democratic and inclusive ways, and to produce research outputs that are aligned with the target audience's needs and priorities.

## 1 INTRODUCTION

The UN Convention on the Rights of the Child^1^ articulated an ambitious ideal: that children have the right to be heard in all matters that affect them. Since publication of the Convention, there has been a growing re‐conceptualization of young people in research and policy contexts, as active social and political agents whose views and experiences are unique and valuable.^2^, ^3^ This shift in landscape has been paralleled by greater commitment to children and young people's participation in decision making by governments, service providers and researchers.^4^ In the field of research, in particular, there has been a surge of interest in empowering young people to take an active role as *co‐actors* in the process, rather than being passive ‘subjects'.^5^, ^6^, ^7^, ^8^ Central to this participatory paradigm is the notion of returning ‘ownership' of the research to participants, and an understanding of research as a process to which both the researcher and the ‘researched' contribute.^9^, ^10^

Co‐production can be defined as a model in which ‘researchers, practitioners and the public work together, sharing power and responsibility from the start to the end of the project, including the generation of knowledge'.^11^ It is a framework grounded in principles of participation, inclusion and autonomy.^12^ Co‐producing research with young people means ensuring that their voices are heard and incorporated throughout, a process that is assumed to hold potential for generating research that is richer, more relevant and better tailored to the needs of the target group.^13^, ^14^, ^15^, ^16^ Even though we still lack systematic evidence on the effects of co‐production, several case studies have documented the benefits of involving young people in research, including facilitating recruitment, producing better research tools,^17^, ^18^ establishing more relevant outcome measures^19^ and generating richer data.^20^

These assumptions and benefits, however, are entirely dependent on *how* the co‐production is implemented. Indeed, as co‐production grows in popularity, so grows the recognition that it represents an ethically and pragmatically complex ideal.^21^, ^22^, ^23^, ^24^ Concerns about this ideal range from practical considerations, such as the need for additional resources to carry out such collaborative work, to more substantive issues, such as potential tokenism and the politics of disagreement when young people's preferences clash with those of the researchers'.^25^, ^26^, ^27^ Young people's involvement, moreover, requires researchers to confront an academic culture influenced by a view of children as ‘unfinished adults',^28^ who lack both rationality and moral agency, and who must be protected from the interests of academic institutions.^29^ Both the practical and the substantive concerns indicate the importance of structured guidance on how to thoughtfully and effectively design a co‐production model of research with young people.

An increasingly common method of implementing co‐production with young people in health research is through advisory groups that include patients, research participants and members of the public. In 2006, the NIHR Clinical Research Network created their first young people's advisory group (YPAG) in Liverpool^30^ to address important challenges with designing and conducting paediatric trials. Since then, numerous YPAGs have been set up, as well as a number of worldwide consortiums, such as the International Children's Advisory Network (iCAN).^31^ Some YPAGs play a more consultative role (for example, improving the quality of information sheets), whereas others take on a more active, collaborative role in shaping the research. For example, they may collaborate with researchers in setting priorities for research, developing tools, writing, etc

There is, however, a lack of practical guidance in the academic literature from researchers who have designed and run young people's advisory groups aligned with a co‐production model. The guidance we present here is grounded in our own experience with the Oxford Neuroscience, Ethics and Society Young People's Advisory Group (NeurOx YPAG), founded in April 2017.

## 2 A SHORT BACKGROUND TO THE NEUROX YPAG

The NeurOx YPAG currently consists of 30 young people (15‐18 years old) from a wide range of backgrounds and schools, but with shared interest in ethics and mental health. The group supports research conducted by the Neuroscience, Ethics and Society Research Group at the University of Oxford. Since its foundation, the YPAG has primarily supported a Wellcome Trust‐funded project titled Becoming Good: Early Intervention and Moral Development in Child Psychiatry (BeGOOD), which investigates ethical concerns that the early intervention paradigm might pose for young people with and without mental health diagnoses.^32^ The YPAG is available to support every stage of research, from refining research questions, to designing materials and research tools (eg, interview guides, digital resources), recruiting, analysing results and disseminating. Within BeGOOD, the group has supported four empirical studies to date.

We acknowledge that the term ‘advisory' does not clearly characterize the role NeurOx YPAG members play in the BeGOOD project, which is that of ‘co‐producers' rather than ‘advisors'. However, we chose to use ‘YPAG' because it is a standard term used for groups where children and young people are involved in shaping research.

The YPAG has become a resource for the Department of Psychiatry and the Health Biomedical Research Centre at the University of Oxford. The group has also supported external research and engagement projects in UK academic and non‐academic institutions, and in international settings (eg, the youth dissemination campaign for the Lancet Commission on Global Mental Health and Sustainable Development^33^ and the BBC *Tomorrow's World* episode on chatbots^34^). Members have joined interview panels for recruitment of public engagement staff at the University. Finally, the group keeps an active social media presence and has presented at conferences and events.

To further extend reach and impact, we have worked to integrate the NeurOx YPAG into various national and international consortia, including GenerationR Alliance,^30^ the European YPAG network^35^ and iCAN, all of which provide useful platforms for training, as well as experience and resource sharing.

## 3 THE NEUROX YPAG MODEL

**Figure 1:**
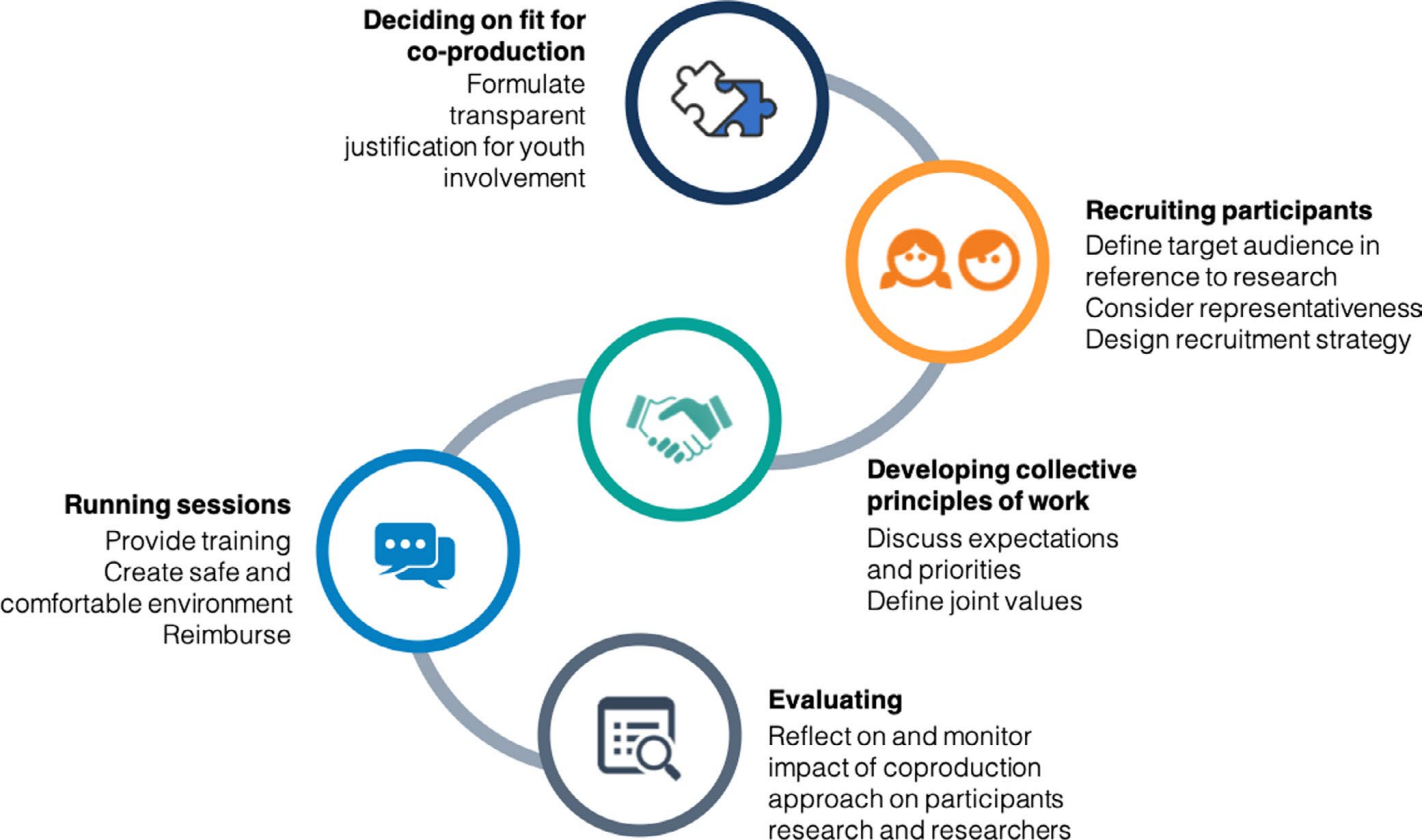
Different steps involved in setting up and working with a YPAG aligned with a co‐production model

The NeurOx YPAG model is summarized in Figure 1. Please note that a number of additional resources, including templates of recruitment materials, activity schedules, assessment questionnaires, consent forms etc, can be accessed on the group's webpage, https://begoodeie.com/ypag-resources/. In what follows, we discuss the different stages of the model in more detail.

### 3.1 Deciding on the fit for co‐production

Formulating a substantial and transparent justification for young people's involvement in research is a fundamental step towards an effective co‐production process. However, a co‐production model of research is not for everyone: there needs to be some theoretical alignment with the research approach. Like other scholars, we do not claim that the co‐production approach is necessarily ethically and scientifically superior to other types of research^7^, ^36^, ^37^, ^38^, ^39^; the decision to involve young people, in particular, should engage both ethical and practical reflection.^40^

Arguably, the most important ethical dimension is careful analysis of whether the benefits of young people's participation outweigh potential harms.^38^, ^40^, ^41^, ^42^, ^43^ For example, the commitment to give voice to youth with particular vulnerabilities, such as personal or family experience of mental health issues, needs to be balanced against the risk of causing harm such as by exposing them to distressing information. The practical dimension should include systematic and thorough evaluation of where in the various stages of research a co‐production approach is most relevant, and can be conducted in a way that is meaningful and impactful.

Some might argue that co‐production requires involvement and engagement in all phases of the research.^44^ However, we support a more flexible definition, where the extent of young people's involvement might vary at different stages of the research, following practical constraints and epistemic limitations. Co‐production should not only focus on the extent of young people's involvement, but also on the quality of their participation.^45^ For instance, in some of our research studies young people were best placed to develop novel methods targeted to their peers (eg, using smartphones), whereas we considered it more appropriate for the researchers to conduct statistical modelling, which would have required young people to undertake extensive training. On the other hand, a co‐production approach with young people should not come to mirror a ‘tick box exercise',^37^ whereby only limited consultations are undertaken, in some cases primarily to fulfil funders' and academic requirements. The discussion surrounding where, how and how much young people are co‐producers in a study is an important one, and should ideally be incorporated into the co‐production process itself, and undertaken with the group from the inception of the research.

### 3.2 Recruiting YPAG Participants

#### 3.2.1 Whom to select?

The target audience for an advisory group must be decided with reference to the research interests, and in many cases, it is advantageous for the characteristics of the advisory group to closely match that of the research population. We acknowledge that young people interested in this type of engagement are unlikely to be fully representative of a larger group^46^; however, efforts can be made to increase the diversity of the advisory group at the outset. Indeed, ‘selective patient and public involvement'^47^ can lead to biases in research priorities and outputs that overly represent the interests of specific sub‐groups.

Knowledge of ‘selective' involvement can also motivate advisory group recruitment targets. In our YPAG, it was important to try to include socially marginalized young people or those with special needs. Such individuals disproportionately access and/or require mental health services, but they have been consistently excluded from research and involvement opportunities in health research more generally.^48^, ^49^, ^50^, ^51^, ^52^ It is also important to keep in mind that some young people engage in part‐time work or other extra‐curricular activities and therefore may be constrained in their ability to take part.^53^ Flexible scheduling can be offered to these participants. Additional support can also be offered to those who might not have some skills required for participation, and different roles can be suggested to participants with different profiles. For example, we invited two YPAG members who were talented writers, but at first felt anxious about participation in group discussions, to form a Writing Committee responsible for blogging about group activities.

#### 3.2.2 The YPAG application process

Our application followed a two‐fold procedure. First, adolescents from a range of schools were invited to apply by filling in an online form.^54^ This form included questions about their motivation to take part in the group, their attitudes with regard to an ethically relevant issue (ie using gene editing to enhance healthy humans) and whether they had any first‐hand experiences with mental health services. Applicants' reasons for joining included interest in the research topic, personal experience with mental health services, a desire to have their voice heard and future career planning. Only very few applicants had taken part in research advisory groups in the past, but about half of the applicants had experience in other group projects such as school debating or volunteer projects. A majority of applicants had personal experience of mental health challenges—either first‐hand or through a close friend or family member.

Second, applicants were invited to a workshop where they took part in a number of small‐group activities (eg, discussing a case study on disclosure of genetic test results to family members) and were given space to ask questions about the project. This gave applicants a ‘taste' of what the YPAG would be like, which helped them determine whether the group would be suitable to them.

Through both stages, motivation to join and engage with our research themes was our key selection criterion, following previous evidence that participatory research can be disrupted when young people feel compelled to get involved or interpret the sessions as ‘schoolwork'.^21^ We also ensured that the group included young people with first‐hand experiences of mental health difficulties, a group who has been traditionally excluded from setting the agenda of ethics research in mental health.

Clearly, when it comes to recruitment there is no one‐size‐fits‐all, and our recruitment procedure cannot simply be applied to any research project. We believe that researchers should design a strategy that allows them to select participants that will most benefit the group—and *from* the group—based on their experience and motivation, while keeping in mind issues of representativeness.

## 4 DEVELOPING COLLECTIVE PRINCIPLES OF WORK

A key stage in setting up an advisory group is the development of collective principles of work. In our group, we dedicated our initial meeting to discussing expectations and priorities and to collectively draft a ‘contract' that reflected our joint values. We agreed that our work should follow principles of responsibility, responsivity and transparency, empathy and acceptance, and confidentiality. Table 1 provides brief descriptions of the pragmatic commitment that each of these principles entailed for participants and facilitators.

**Table 1:** Values and associated commitments agreed upon by NeurOx YPAG youth and facilitators

Principles	Participants	Facilitators
Responsibility	Attending most group meetings Participating actively in the YPAG activities during and in‐between sessions	Making the sessions engaging and entertaining Providing training as needed Providing subsistence, pro‐bona and transport reimbursement for each meeting
Responsivity and transparency	Communicating effectively Providing honest feedback	Communicating effectively Incorporating and recognizing YPAG members' contributions
Empathy and acceptance	Being respectful and accepting of each other's opinions Giving space for everyone to participate	Creating a safe and comfortable space for participants to share ideas Ensuring that everyone in the group has a chance to have their voice heard
Confidentiality	Keeping any personal narratives shared in the group strictly confidential	Keeping any personal narratives shared in the group strictly confidential

This critical stage reinforces the co‐constructed nature of the group and its commitment to deliberative democratic principles such as reciprocity.^55^ The co‐signed contract provides helpful benchmarks for evaluation and facilitates commitment and accountability. Making it flexible allows us to adapt to changes in circumstances or any potential inconsistencies between the ideal and the practical.

## 5 RUNNING A MEETING

To facilitate effective participation, it is often necessary to train the group on research methods, data protection and some of the theoretical background of the research. The goal is not to make young people ‘experts' in the research area, but to provide participants with enough information to facilitate their meaningful contribution to the project. Indeed, Thompson et al^56^ warn researchers of the risk of overtraining or ‘professionalizing' members of advisory groups, who might then cease to represent ‘the public'.

It is also essential that facilitators are equipped with the right skill set to provide a comfortable and engaging environment for the group, and that participants understand it to be a non‐judgmental space to collectively generate ideas, comment and criticize. This aligns to the value that the co‐production model places in the different kinds of expertise, particularly researchers' academic expertise and participants' experiential expertise in the production of knowledge.^57^, ^58^

Arguably, the greatest challenge that may arise from co‐producing research with young people refers to their need to be protected from harm.^59^, ^60^, ^61^ It is important that facilitators develop a child protection protocol, tailored to the needs and potential vulnerabilities of their particular group. For example, in the NeurOx YPAG, partially because many participants had first‐hand experience of mental health difficulties, we invited a clinically trained psychologist to attend our initial session. We also encouraged participants to notify the session facilitator in case they felt distressed, and made it clear that they could choose *not* to participate in discussions/data collection if they did not feel comfortable talking about certain topics. Having at least two facilitators present in each session and holding contact information of YPAG members' parents/guardians might also be helpful measures. Facilitators should also have appropriate reporting processes in place, following national and local guidelines, if any serious risk of harm is identified.

In terms of session structure, we find it helpful to keep a similar schedule for each meeting, with a mix of small and large group activities.^54^ We find that our co‐production process works most effectively when the group is presented with open‐ended activities and questions, which gives YPAG members greater autonomy and agency, instead of highly structured tasks. For example, when the group co‐designed the Interview Guide for a study on young people's moral attitudes towards genetic testing for Alzheimer's disease, we gave a brief overview of the theoretical background and our outcome variables of interest and then invited the group to formulate activities and questions to best capture that information. A short description of this and other sessions is available at https://begoodeie.com/ypag/.

It is important to note that group members are likely to vary in terms of how much time they wish to dedicate to the group, and how they would like to contribute. In our group, one way we accommodate these differences is by taking a layered approach, where in addition to regular meetings, all YPAG members are offered a number of optional opportunities. This includes speaking at conferences, co‐writing manuscripts and engaging with research participants. This approach allows for the group to be tailored to participants' skill sets and individual interests. It also aligns with our commitment to involve group members in deciding the extent and content of their involvement in co‐production.

Facilitators must also be prepared to manage potential differences in opinions among young people, or between YPAG members and researchers, as well as situations where young people's feedback cannot be incorporated. For example, when planning a mental health awareness campaign, YPAG members suggested launching a social media challenge that encouraged young people to post videos of themselves waking someone up, which would act as a metaphor to increasing awareness. Even though we thought that was a powerful metaphor, we were concerned that it could violate the privacy of those ‘woken up' if young people recorded and posted the videos without their consent. When such discrepancies arise, we believe that the most helpful approach is to dedicate time to discuss the issue, and to be open and transparent about any concerns both parties might have.

When research results are published, it is important that the YPAG's involvement is noted, for example in the body of the paper or acknowledgements. In some instances, however, their involvement warrants (co)‐authorship of the relevant outputs. This occurs when YPAG members have made substantial contributions to the research concept and design, data collection, and/or analysis and interpretation of results. In these cases, they would also participate in drafting the article or critically revising it, and approving the final version. This arrangement is consistent with the general guidance from the International Committee of Medical Journal Editors (ICMJE)^62^ on academic authorship. NeurOx YPAG members have recently co‐authored a manuscript on the ethics of using chatbots in mental health support,^63^ and the present manuscript is co‐authored by Ed Goundry‐Smith, who contributed a section on his first‐hand experience and critically appraised the draft for submission. Overall, it is important that these measures are agreed upon with the group and that this is done early in each research project.

Finally, it is important to reimburse YPAG members for their work. The payment should not only be a fair return to their efforts but also conform to cultural and social norms.^59^ At the NeurOx YPAG, each member receives a £25 gift voucher for each half‐day meeting attended, which is consistent with guidelines developed by INVOLVE.^64^

## 6 EVALUATING IMPACT

Evaluation of both participants and researchers is an integral part of critically running a YPAG. We periodically ask participants to fill in anonymous assessment questionnaires and indicate what they consider to be priorities for the group moving forward.^54^ Understanding the first‐hand experiences of YPAG members helps us ensure that we are offering the right level of information, training, support and compensation. For example, following feedback from YPAG members, we have made changes to the structure of the sessions, favouring ‘active' tasks over passive activities such as reading or listening to a talk, and small over large group discussions.

**Figure 2:**
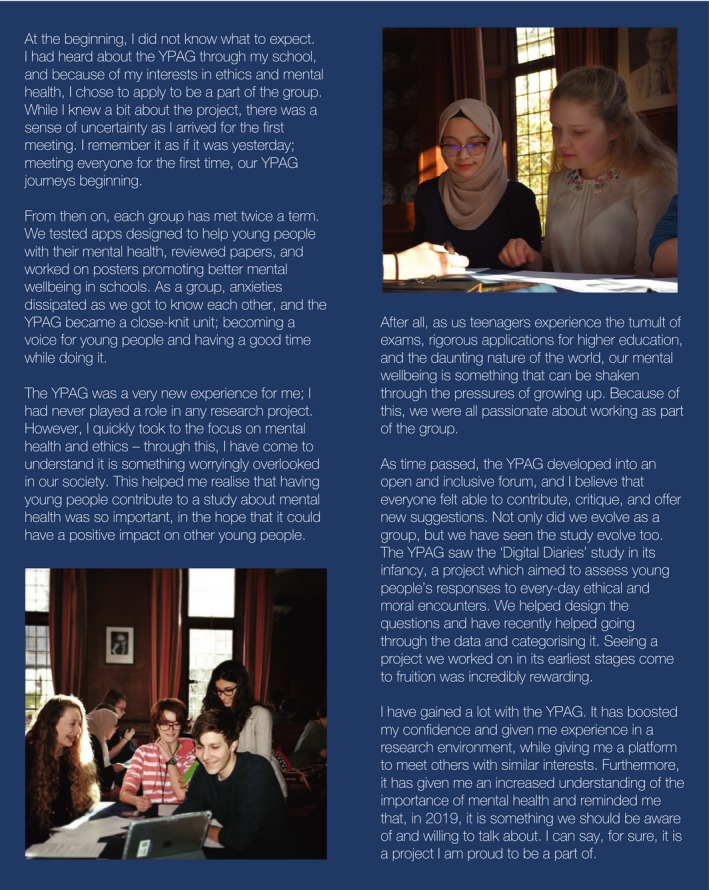
A first‐hand account of a NeurOx YPAG member

We also ask participants to reflect upon the learning and skills they might have gained from participating and any impact on academic and personal development. It is not a given that young people benefit from engagement schemes^5^, so this helps us assess the impact of their involvement in a systematic way. Overall, NeurOx YPAG members indicated that their participation helped them gain both technical and soft skills. The former includes knowledge on research methods and the research theme (eg, ‘[I learnt] how to be analytical with research'). The latter includes confidence, openness and teamwork (eg, ‘[I learnt] to listen open mindedly to other people's opinions'). In Figure 2, Ed Goundry‐Smith offers a first‐person account of his experience as a member of the group.

In addition to monitoring the impact of the project for YPAG members, a continuous assessment and documentation of how the project has changed the research is also essential. Because we work in close partnership throughout the research process, it is difficult to imagine what the research would have looked like had the young people not been involved. Below are specific examples of how the group's involvement has impacted different stages of the process.

Following feedback from the YPAG we have:

1. Shifted our research focus from the ethics of predictive genetic testing to the ethics of predictions based on digital footprints, which the group deemed more relevant to their daily lives.
2. Adopted peer‐led interviews as a research tool, whereby participants take turns asking pre‐defined questions to each other (drawn from a pile of flashcards), rather than the traditional researcher‐youth set‐up. Feedback from piloting interviews suggested that this type of set‐up, which resembles a real‐life conversation between peers, is comfortable and engaging for young people and gives them a greater sense of agency.
3. Developed digital games to be used as tools to collect empirical data, which the group considered to be a highly engaging method. For example, the group developed the initial concept of a digital role‐playing scenario whereby participants take the role of customers of a company that offers predictive testing for mental health, which we are currently using as empirical tool in a study titled ‘What lies ahead?'. Details of one of our brainstorming sessions on games are available at https://begoodeie.com/ypag/ypag-blog-1/apps-and-games/.
4. Implemented more effective recruitment strategies, leveraging online platforms.

A thorough evaluation of the impact of the project on the youth, researchers and the research is not only essential for internal monitoring purposes, but also contributes relevant evidence to the scarce body of literature on the impact of youth involvement with research (but see 65, 66 for notable exceptions).

## 7 CONCLUSION

The increasing pressure from funding bodies and the academic community for researchers to adopt participatory methods poses the risk that they will do so in an uncritical manner.^38^, ^39^ The step‐by‐step guide we present here emphasizes the importance of taking a reflective and reasoned stance throughout the whole process. First, we acknowledge that co‐production and advisory groups are not necessary in every project and invite researchers to carefully evaluate whether this model fits their own aims. We encourage researchers to be reflective during the selection process and the running of the sessions, ensuring that different interests and voices are represented. Finally, we highlight the importance of on‐going evaluations on the impact of the group on both the young people and the research, and reflections upon whether the group is mutually beneficial, and genuinely empowering for young people rather than reinforcing patronising assumptions about their vulnerability. Adopting an open and reflective perspective from beginning to end can increase researchers' capacity to engage young people in ways that are meaningful, democratic and inclusive.

## CONFLICT OF INTEREST

The authors declare no conflict of interest.

## DATA AVAILABILITY STATEMENT

Data sharing is not applicable to this article as no new data were created or analysed in this study.
